# Enhancing dietary adherence among African-American adolescents: the role of parenting styles and food-related practices

**DOI:** 10.3389/fnut.2024.1254338

**Published:** 2024-05-09

**Authors:** Azam Ardakani, Lillie Monroe-Lord, Dorothy Wakefield, Chimene Castor

**Affiliations:** ^1^College of Agriculture, Urban Sustainability and Environmental Sciences, University of the District of Columbia, Washington, DC, United States; ^2^Department of Nutritional Sciences, Howard University, Washington, DC, United States

**Keywords:** parenting styles, food-related parenting practices, MyPlate, African-American, parent-adolescent dyads, dietary guidelines for Americans

## Abstract

**Introduction:**

Parenting styles (PSs) and food-related parenting practices (FPPs) play a crucial role in shaping adolescent eating behavior. This study aimed to investigate the relationship between the different PSs and FPPs of African-American families and the frequency of consumption of MyPlate food items by adolescents based on recommendations from the Dietary Guidelines for Americans (DGA).

**Methods:**

This study used a cross-sectional design. Data collection was conducted using Qualtrics through an online survey of 211 African-American parents and their adolescents aged 10–17-year-old. Adolescents completed the Youth and Adolescent Food Frequency Questionnaire to assess their dietary behavior, while parents filled out the survey to identify the degree of PSs (i.e., authoritative, authoritarian, setting rules, and neglecting) and FPPs (i.e., monitoring, reasoning, copying, and modeling). Spearman’s rank correlation coefficient, Wilcoxon rank-sum test, and stepwise logistic regression were performed to determine the answers to the research questions.

**Results:**

For fruit consumption, authoritative parenting significantly reduced the likelihood of adherence to DGA, while authoritarian, monitoring, and reasoning practices increased it. Female adolescents were more likely to meet fruit intake recommendations, with a similar positive impact observed for those whose parents had above high school education. In vegetable intake, authoritarian and monitoring practices positively impact on adherence to DGA, whereas setting rules had a detrimental impact. Being in a married household also increased vegetable intake DGA adherence. For grain consumption, reasoning was a significant positive predictor, while setting rules negatively impacted adherence. Dairy DGA adherence was positively impacted by monitoring and copying practices, but negatively impacted by female gender. Protein intake showed a positive association with reasoning and parental education.

**Discussion:**

Our findings confirm the importance of parenting in developing desired eating behaviors among African-American adolescents. The results of this study can be used to develop culture-based nutritional education programs for parents and youth.

## 1 Introduction

Adolescence is a vital period for adopting healthy eating behaviors and a balanced diet, key for acquiring essential nutrients required for the physical and mental development of adolescents. This stage also sets the foundation for sustaining dietary preference into adulthood (1). Currently, there is a significant concern regarding adherence of adolescents to the daily minimum amount (DMA) of MyPlate food groups, based on the Dietary Guidelines for Americans (DGA) (2). Designed by the United States Department of Agriculture (USDA), MyPlate provides a comprehensive healthy eating habit guide for five major food groups, i.e., fruits, vegetables, grains, dairy, and protein (3). The DGA provides information about dietary pattern and reports the recommended DMA for each MyPlate item based on age and sex.

Although adolescents have become more independent in their food choices owing to the influence of their peers, the impact of the family environment is significant (4). Parenting styles (PSs) and food-related parenting practices (FPPs) affect the development and improvement of dietary habits (5–7). Developmental psychologists identify different PSs, including authoritative, authoritarian, permissive, and neglectful styles. These styles are classified based on the degree of parental responsiveness and demandingness, which affect the regulation of norms and behaviors of adolescents (8, 9). In addition, FPPs are considered separate constructs derived from PSs, and are goal-oriented behavioral strategies that parents use during different eating situations, such as meal or snack times, which can impact the diet and weight status of their children (10). These FPPs, which encompass a variety of approaches, including availability, accessibility, responsibility, monitoring, modeling, encouragement, restriction, mealtime structures, setting rules and expectations, and pressuring to eat, play a crucial role in shaping adolescents’ dietary behaviors (7, 11, 12).

Notably, research has shown that the adoption of higher monitoring and modeling FPPs is associated with increased consumption of fruits and vegetables among adolescents from diverse racial backgrounds (13). Higher-reasoning FPPs and authoritative parenting styles have been shown to decrease the consumption of unhealthy snacks, while setting numerous rules has been linked to increased consumption of unhealthy snacks due to the perceived threat to adolescents’ autonomy (13). Parents’ eating habits and FPPs directly impact their adolescents’ eating habits, with parents who encouraged fruit and vegetable consumption tending to have adolescents who consumed more items from these groups. However, parents who exert control to limit junk food and sugary drink consumption may inadvertently contribute to the increased consumption of unhealthy foods among adolescents (14). Furthermore, research has demonstrated that autonomy-supportive and intrinsic motivational practices of parents are associated with improved fruit and vegetable intake and reduced consumption of sugar-sweetened beverages among boys. Structured parenting practices showed positive effects on dietary behaviors for both sexes, whereas controlling and autonomy-supportive practices had indirect effects on boys’ dietary behaviors through motivation (15). The present study extends this existing body of knowledge by specifically investigating the influence of PSs and FPPs on the comprehensive dietary patterns of African-American adolescents, a focus that is notably absent from current literature. The impact of PSs and FPPs may vary across races and ethnicities, particularly among minority groups. For instance, among African-American families, an authoritarian parenting style characterized by rigidity and restriction has been identified as a hindrance to adolescents’ self-efficacy and healthy eating habits (16, 17). A study with a small sample size of 14 participants found that African Americans predominantly utilized modeling practices and setting rule styles as their dominant PSs and FPPs (18). Furthermore, monitoring practices have been shown to positively influence the consumption of fruit and vegetable by African-American adolescents (13). However, limiting the consumption of unhealthy foods among African-American adolescent boys has been associated with a higher risk of being overweight or obese (19). While previous studies have explored the impact of specific PSs and FPPs on limited food items or food groups, the current study aims to expand this investigation by considering all of MyPlate food items consumption. This study also intends to comprehensively examine four PSs (authoritative, authoritarian, setting rules, and neglecting) and four FPPs (monitoring, reasoning, copying, and modeling). Monitoring and reasoning are direct, communicative strategies that encourage awareness and understanding of healthy eating choices (10). Copying and modeling leverage parental behavior as a template for healthy eating, promoting learning through observation and imitation (7).

Two theoretical frameworks, the Social Cognitive Theory (SCT) and Family System Theory (FST), are foundational to our understanding of the relationships between PS, FPP, and eating habits among adolescents. These two frameworks offer valuable insights into the complex interplay among family dynamics, individual cognition, and sociodemographic factors that shape dietary behaviors. SCT focuses on the reciprocal influence of individual experiences, behaviors, the actions of others, and environmental factors on individual health behaviors (20). SCT has also been frequently used to evaluate the effects of influential factors such as FPPs, PSs, and demographic characteristics on the eating behaviors of adolescents (21). Self-efficacy as a construct of SCT can help us understand how we can enforce healthy behaviors among adolescents by applying PS and FPPs (22). By enhancing adolescents’ self-efficacy through positive reinforcement, parents can increase the likelihood of their children engaging in healthy behaviors and making healthy food choices. In addition, FST highlights the importance of the family system in explaining individual behavior (23). Based on FST, the functionality and behavior of individuals are related to their interactions with family members, with each individual in a family playing a defined role in interactions with other family members (24). Consequently, any change in the family structure, or even in one of the family members, can change the behavior of other family members (25). For instance, a warm and supportive PS is correlated with improved adolescent eating behavior (17, 26).

The number of studies examining the effect of the family environment on the eating habits of adolescents, especially among minorities such as African-American adolescents, is limited. This study not only assesses the impact of various PSs and FPPs on the consumption of DGA-recommended DMA for all MyPlate items but also aims to explore differences in eating habits between adolescents who meet and those who do not meet the guideline. The findings of this study will help specialists develop interventions or educational sessions to improve the eating habits of African-American adolescents.

## 2 Methods

### 2.1 Research design, participants, and procedure

Prior to data collection, the Institutional Review Board responsible for overseeing human subject research at the University of the District of Columbia approved this study. This study was designed as a cross-sectional investigation in which data were collected from 211 African-American parent-adolescent dyads. Participants were recruited through email invitations sent via Qualtrics. Participants were provided with a link to an online survey that included both parental consent and adolescent assent forms. Each parent and his/her adolescent completed the survey through a single integrated survey link. The adolescents completed the first part of the survey, which focused on their dietary habits, and their parents completed the second part, which focused on their PSs and FPPs. The inclusion criteria required participants to (1) self-identify as African American, (2) be parents or legal guardians of adolescents aged 10–17 years, and (3) reside in the United States at the time of the study. The exclusion criteria disqualified potential participants if they: (1) did not identify as African-American, (2) did not have children or legal guardianship of children within the specified age range, or (3) resided outside the United States. If participants did not meet the inclusion criteria, the survey was automatically terminated.

### 2.2 Survey

The survey used various tools for gathering comprehensive data. For this study, we focused on a specific portion of the collected data that pertained to participants’ responses regarding sociodemographic attributes, consumption of various MyPlate food items (as reported by adolescents), and assessment of PSs and FPPs (as reported by parents).

#### 2.2.1 Demographic characteristics

The study collected demographic characteristics of the participants, including the age and sex of the adolescents, as well as the age and sex of their parents. Education level, household income, marital status, and the relationship between adult and adolescent participants were also recorded. Descriptive statistics, such as percentages, means, and standard deviations, were calculated to summarize demographic characteristics.

#### 2.2.2 MyPlate food item consumption

To assess adolescent food consumption, the 2012 Youth Adolescent Food Frequency Questionnaire (YAQ FFQ/FFQ) was administrated, detailing the type, frequency of intake, and portion size of each food (27), which was subsequently analyzed to obtain data regarding various MyPlate food items. These items were selected because they reflected the recommended dietary guidelines for a healthy and balanced diet (27, 28). Adolescents reported their consumption retrospectively for over the past year. The specific procedures used to retrieve MyPlate food items from the FFQ reports are detailed in Table 1. Furthermore, to identify whether adolescents met the recommended DMA for each MyPlate food item, we referred to the guidelines provided by the DGA and the USDA handbook (28). These guidelines specify the recommended DMA for each food item based on the age and sex of the adolescents. By comparing the reported consumption of each MyPlate item with the corresponding DMA, we determined which adolescents met the recommended intake and those who fell short of it.

**Table 1:** The DMA and composition of MyPlate food item for adolescents by age and gender.

Fruit			
Female		Male	
9–13 years old	14–18 years old	9–13 years old	14–18 years old
1½ cups	1½ cups	1½ cups	2 cups
Bananas; apples; grapes; applesauce; cantaloupe, melon; watermelon; oranges; strawberries; blueberries; pear; peaches, grapefruit; plums, apricots; pineapple; apple juice; orange juice; and other 100% fruit juices; raisins; mixed dried fruit/trail mix.			

Vegetable			
Female		Male	
9–13 years old	14–18 years old	9–13 years old	14–18 years old
1½ cups	2 ½ cups	2 cups	2 ½ cups
Tomatoes; tomato juice; broccoli; green beans; cauliflower; peas; mixed vegetables; corn; spinach, raw as in salad; collard greens/kale/cooked spinach; yam/sweet potatoes; zucchini, summer squash; eggplant; green/red/yellow peppers; carrots, cooked; carrots, raw; lettuce/tossed salad; celery; coleslaw; onion rings, cabbage; okra; cooked onion, or onion soup.			

Grain			
Female		Male	
9–13 years old	14–18 years old	9–13 years old	14–18 years old
5 ounce equivalents	6 ounce equivalents	5 ounce equivalents	6 ounce equivalents
Cold breakfast cereal; oatmeal, including instant; other cooked breakfast cereal (e.g., cream of wheat, grits); white bread, pita bread, including toast (not in sandwich); whole wheat or whole grain bread, including toast (not in sandwich); muffin or cornbread; English muffins, bagels, or rolls (include breakfast sandwich); croissant; white rice; brown rice; biscuit; quesadilla not tacos or burritos; corn or flour tortilla- no filling, e.g., pancakes or waffles; French toast; potatoes-baked or boiled mashed.			

Dairy			
Female		Male	
9–13 years old	14–18 years old	9–13 years old	14–18 years old
3 cups	3 cups	3 cups	3 cups
Milk, chocolate, or other flavored milk; instant breakfast drink; plain/low-calorie yogurt; regular yogurt sweetened with fruit or other flavoring cheese, cream cheese, cottage or ricotta cheese.			

Protein			
Female		Male	
9–13 years old	14–18 years old	9–13 years old	14–18 years old
2 serving size	2.5 serving size	2.5 serving size	2.75 serving size
Cheeseburger; tofu, soy burger, hamburger or Sloppy Joe; miso, edamame, or other soy dish; veggie burger; tacos; pizza; burritos; chicken nuggets; beef or pork hot dogs (include corndogs); chicken or turkey hot dogs or sausage; chicken or turkey as a mixed dish (e.g., stir fry or soup); chicken or turkey as main dish; fish sticks, fish cakes or fish sandwich; dark meat fish as main dish, e.g., tuna steak, salmon sardines, swordfish; other fish as main dish, e.g., cod, haddock, halibut; shrimp, lobster, scallops; beef, pork or lamb as a mixed dish (e.g., stir fry or stew); beef (steak, roast) or lamb as main dish; liver; pork, ribs, or ham as main dish; meatballs or meatloaf; eggs; sausage (beef/pork); peanut butter sandwich, chicken or turkey sandwich, roast beef sandwich; salami, bologna, ham or other deli meat; beans or lentils (include baked beans); tuna sandwich; peanut; other nuts.			

According to the DGA, DMA is assessed separately for two age groups: 9–13 years old and 14–18 years old. For fruit consumption, DMA is recommended at 1 ½ cups for both girls and boys aged 9–13 years old, and 2 cups for boys aged 14–18 years old. In terms of vegetable intake, the DMA is 1 ½ cups for girls and boys aged 9–13 years old, and 2 ½ cups for both sexes in the 14–18 age group. For grain consumption, DMA was set at 5 ounce equivalents for all adolescents aged 9–13 years old, and 6 ounce equivalents for those aged 14–18 years old. The DMA for dairy is consistent, at three cups for adolescents of all ages. Finally, the DMA for protein varies with sex and age: two serving sizes for girls aged 9–13 years old, 2.5 serving sizes for girls aged 14–18 years old and for boys aged 9–13 years old, and two ¾ serving sizes for boys aged 14–18 years old (28). Table 1 summarizes the DMA by the age and sex of the adolescents.

#### 2.2.3 PSs and FPPs

The FPP questions were used from Monroe-Lord et al. (13), who explored the impact of FPP on the eating behavior of adolescents among African-American families (18). In addition, the 85-item Comprehensive General Parenting Questionnaire was used to identify PSs (29). Further details about the parent part of the survey are available in a study by Gunther et al. (30). Responses were collected using a 5-point Likert scale ranging from “strongly disagree” to “strongly agree” with “sometimes” as the mid-point or from “never” to “always” featuring “neutral” as its mid-point. Specifically, the FPPs measured in this study included monitoring, reasoning, copying, and role-modeling. Monitoring is defined as parent consistently overseeing their children’s food intake, with a focus on the type and quantities of food consumed. Reasoning is defined as parents imparting knowledge to their children about the benefits of nutritious foods and guiding them toward establishing healthy eating habits. Copying is defined as when parents intentionally or unintentionally lead their children to mimic their eating habits. Modeling is defined as parents actively demonstrating healthy eating habits to inspire their children to adopt similar behavior through observational learning. PSs were categorized as authoritative, authoritarian, setting rules/expectations, and neglecting. Authoritative is defined as when parenting combines attentive, empathetic engagement with consistent, fair guidance and support for a child’s autonomy. Authoritarian is characterized by strict enforcement of rules, limited emotional responsiveness, and a strong emphasis on obedience and control over a child’s actions and feelings. Setting rules is defined as a parenting approach focused on setting clear expectations and rules, with an emphasis on obedience and the parent’s authority in the family dynamic. Neglecting style is defined as characterized by inconsistency in enforcing discipline and a lack of follow-through on consequences. The more details of how the FPPs and PSs were identified and named are reported in our earlier study (31). Each parent was assigned a score for FPP and PS based on their responses to the survey questions.

### 2.3 Statistical analysis

All statistical analyses were performed using SAS version 9.4 (SAS Institute, Cary, NC, United States). In this study, the independent variables considered were PSs and FPPs, whereas the dependent variable was meeting the DGA criteria. Meeting the DGA were evaluated based on the frequency of consumption of MyPlate items, measured as serving size per week. Three statistical tests were used: Spearman’s correlation coefficient, Wilcoxon rank-sum test, and stepwise logistic regression analysis. Spearman’s correlation coefficient was used to assess the strength and direction of the relationships between PSs, FPPs, and eating habits. The Wilcoxon rank-sum test, a non-parametric test, was used to compare the differences in eating habits between the two groups of adolescents: those who met or did not meet the DGA criteria for each food item. Because we examined the relationship of each food group with the four PSs and four FPPs, we have indicated the adjusted *p*-value (Bonferroni adjustment, α/8) significance in Tables 2, 3. Five separate stepwise logistic regression models (one for each food group) were used to identify the predictors of meeting the DGA criteria (Yes/No). The potential covariates included FPPs and PSs as well as the following dyad demographic parameters: family income (under $45,000/ $45,000 and above), parent and adolescent sex (Female/Male), parent age (under35 years/ 35 years and above), adolescent age, marital status (married/not married), and parent education (high school graduate or not). Stepwise regression parameters were set so that a *p*-value of 0.10 was required to stay in the model. Results were considered statistically significant if the *p*-values were < 0.05.

**Table 2:** Demographic characteristics for the African American parent-adolescents dyads.

Characteristic	*N* (%)
Adolescent sex	
Male	87 (41.23)
Female	124 (58.77)
Parent sex	
Male	63 (29.87)
Female	148 (70.14)
Adolescent age	
10–13	82 (38.86)
14–17	129 (61.14)
Parent age	
18–25	42 (19.91)
26–34	66 (31.28)
35–54	98 (46.45)
55–64	3 (1.42)
≥65	2 (0.95)
Parent education	
Not completed high school	80 (37.91)
High school diploma or GED	12 (5.69)
Some/4-year college, technical school, or advanced degree	119 (56.40)
Household income (USD)	
0–$44,999	85 (40.28)
$45,000–$84,999	70 (33.18)
$85,000 or more	49 (23.22)
Prefer not to answer	7 (3.23)
Marital status	
Married	102 (48.34)
Single (includes divorced, never married, and widowed)	109 (51.67)
Relationship with adolescent	
Parent (includes step/foster parent)	173 (81.99)
Other caregivers	38 (18.01)

**Table 3:** Relationships between the consumption of different MyPlate food items and PSs and FPPs.

	Fruit		Vegetable		Grain		Dairy		Protein	
	*r*^a^	*p*-value	*r*^a^	*p*-value	*r*^a^	*p*-value	*r*^a^	*p*-value	*r*^a^	*p*-value
Parenting styles										
Authoritative	0.04	0.528	−0.02	0.773	−0.01	0.919	−0.10	0.119	0.010	0.887
Authoritarian	0.21	**0.0017**^b^	0.28	**<0.0001**^b^	0.23	**0.0005**^b^	0.18	**0.0078**^b^	0.29	**<0.0001**^b^
Setting rules	0.03	0.6439	−0.06	0.3172	0.66	0.3385	0.14	**0.0381**	−0.07	0.2867
Neglecting	0.07	0.2639	0.08	0.1993	0.08	0.2364	0.14	**0.0381**	0.13	**0.0496**
Food-related parenting practices										
Monitoring	0.26	**0.0001**^b^	0.18	**0.008**	0.20	**0.003**^b^	0.27	**<0.0001**^b^	0.17	**0.011**
Reasoning	0.36	**<0.0001**^b^	0.26	**<0.0001**^b^	0.26	**0.0001**^b^	0.28	**<0.0001**^b^	0.24	**0.0005**^b^
Copying	0.25	**0.0002**^b^	0.23	**0.0006**^b^	0.25	**0.0002**^b^	0.23	**0.0006**^b^	0.25	**0.0003**^b^
Modeling	0.23	**0.0006**^b^	0.11	0.11	0.11	0.11	0.24	**0.0005**^b^	0.11	0.09

a Correlation coefficient.

b Bonferroni adjusted *p*-value is significant.

FPPs, Food-related parenting practices; PS, parenting styles.

## 3 Results

### 3.1 Demographic analysis

A total of 211 adolescents participated in the study, with a mean age of 14.28 years (SD = 2.32). Among them, 129 (61.14%) were between 14 and 17 years old and 124 (58.77%) were female. For the parents’ characteristics, the age distribution was as follows: 42 (19.91%) were between 18 and 25 years old, 66 (31.28%) were between 26 and 34 years old, 98 (46.45%) were between 35 and 54 years old, 3 (1.42%) were–55-64 years old, and 2 (0.95%) were 65 years or older. Most parents were female, accounting for 148 (70.14%) participants. in term of parents’ education, more than half of participants 119 (56.40) were in Some/4-year college, technical school, or advanced degree category. Regarding household income, 85 (40.28%) of families earned less than $45,000 annually. Considering marital status, 109 (51.67%) participants reported being single. The majority of participants (173 (81.99%)) had a parent–child relationship, which included biological, step-, or foster parents. A detailed summary of the sample characteristics can be found in Table 2.

### 3.2 PSs, FPPs, and frequency of consumption of MyPlate food items

Associations between the consumption of different MyPlate food items, PSs, and FPPs were examined. The authoritarian PS alone was significantly positively correlated with the consumption of all MyPlate food items (fruit: *r* = 0.21, *p* = 0.0017; vegetable: *r* = 0.28, *p* < 0.0001; grain: *r* = 0.23, *p* = 0.0005; dairy: *r* = 0.18, *p* = 0.0078; and protein: *r* = 0.29, *p* < 0.0001). Setting rules and neglecting PSs were positively correlated with dairy consumption (*r* = 0.14, *p* = 0.0381 for both PSs). Neglecting PS was significantly correlated with protein consumption (*r* = 0.13, *p* = 0.0496). Authoritative PS did not correlate with any of the MyPlate food items consumed.

Monitoring (fruit: *r* = 0.26, *p* = 0.0001; vegetable: *r* = 0.18, *p* = 0.008; grain: *r* = 0.20, *p* = 0.003; dairy: *r* = 0.27, *p* < 0.0001; and protein: *r* = 0.17, *p* = 0.011), reasoning (fruit: *r* = 0.36, *p* < 0.0001; vegetable: *r* = 0.26, *p* < 0.001; grain: *r* = 0.26, *p* = 0.0001; dairy: *r* = 0.28, *p* < 0.0001; and protein: *r* = 0.24, *p* = 0.0005), and copying FPPs (fruit: *r* = 0.25, *p* = 0.0002; vegetable: *r* = 0.23, *p* = 0.0006; grain: *r* = 0.25, *p* = 0.0002; dairy: *r* = 0.23, *p* = 0.0006; and protein: *r* = 0.25, *p* = 0.0003) were significantly correlated with the consumption of all MyPlate food items. The correlation coefficients between the three FPPs and all MyPlate food items were positive. Moreover, role modeling was significantly and positively correlated with the consumption of two MyPlate food items: fruit and dairy (*r* = 0.23, *p* = 0.0006 and *r* = 0.24, *p* = 0.0005, respectively) (Table 3).

### 3.3 PSs, FPPs, and DGA for the frequency of consumption of MyPlate food items

The relationships between PSs and FPPs and whether adolescents met the DGA recommendations for the consumption of different MyPlate food items were examined. Table 4 shows a comparison of PSs and FPPs between adolescents who did and did not meet the recommended DGA for the consumption of different MyPlate food items. Overall, the percentages of African-American adolescents who met the DGA for the consumption of different MyPlate food items were 81% (protein), 65% (fruit), 56% (vegetables), 40% (grains), and 32% (dairy).

**Table 4:** Comparison of PS and FPPs among adolescents who did and did not consume the DGA-recommended amounts of all MyPlate food items.

		Mean (SD)				
		Fruit	Vegetable	Grain	Dairy	Protein
Authoritative						
Not met DGA		4.13 (0.82)	4.12 (0.74)	4.07 (0.79)	4.04 (0.78)	4.08 (0.75)
	Met DGA	4.07 (0.77)	4.07 (0.82)	4.13 (0.78)	4.21 (0.79)	4.09 (0.80)
	*p* value	0.4551	0.8779	0.6031	0.1209	0.8093
Authoritarian						
	Not met DGA	3.15 (0.97)	3.27 (0.92)	3.31 (0.93)	3.30 (0.88)	3.17 (0.89)
	Met DGA	3.60 (0.89)	3.57 (0.94)	3.63 (0.92)	3.74 (1.00)	3.50 (0.94)
	*p* value	**0.0013**^a^	**0.0101**^a^	**0.0158**^a^	**0.0016**^a^	**0.0305**^a^
Setting rules						
	Not met DGA	4.09 (0.88)	4.14 (0.79)	4.09 (0.87)	4.02 (0.81)	4.14 (0.79)
	Met DGA	4.09 (0.79)	4.05 (0.84)	4.08 (0.76)	4.25 (0.82)	4.08 (0.83)
	*p* value	0.7409	0.4162	0.6053	**0.0293**^a^	0.6704
Neglecting						
	Not met DGA	3.42 (1.23)	3.58 (1.11)	3.54 (1.17)	3.49 (1.13)	3.47 (1.09)
	Met DGA	3.68 (1.10)	3.60 (1.19)	3.66 (1.13)	3.81 (1.19)	3.62 (1.17)
	*p* value	0.1453	0.7459	0.4992	**0.0325**^a^	0.3750
Monitoring						
	Not met DGA	3.18 (0.95)	3.25 (0.92)	3.29 (0.85)	3.28 (0.84)	3.36 (0.77)
	Met DGA	3.56 (0.77)	3.57 (0.78)	3.63 (0.84)	3.74 (0.80)	3.44 (0.88)
	*p* value	**0.0095**^a^	**0.0311**^a^	**0.0083**^a^	**0.0003**^a^	0.4644
Reasoning						
	Not met DGA	3.17 (0.93)	3.32 (0.93)	3.29 (0.86)	3.32 (0.88)	3.16 (0.96)
	Met DGA	3.61 (0.79)	3.56 (0.80)	3.70 (0.82)	3.74 (0.77)	3.52 (0.83)
	*p* value	**0.0006** ^a^	0.0577	**0.0008** ^a^	**0.0009** ^a^	**0.0184** ^a^
Copying						
	Not met DGA	3.24 (0.73)	3.32 (0.75)	3.29 (0.77)	3.27 (0.78)	3.21 (0.62)
	Met DGA	3.50 (0.85)	3.49 (0.86)	3.60 (0.85)	3.71 (0.81)	3.46 (0.85)
	*p* value	**0.0247** ^a^	0.0779	**0.0059** ^a^	**0.0004** ^a^	**0.0467** ^a^
Modeling						
	Not met DGA	3.45 (0.48)	3.62 (0.81)	3.55 (0.83)	3.51 (0.80)	3.48 (0.73)
	Met DGA	3.71(0.79)	3.62 (0.83)	3.72 (0.80)	3.86 (0.82)	3.65 (0.84)
	*p* value	**0.0222** ^a^	0.6560	0.0979	**0.0007** ^a^	0.1315

a Bonferroni adjusted *p*-value is significant.

DGA, Dietary Guidelines for Americans; FPPs, food-related parenting practices; PS, parenting styles; SD, standard deviation.

There were no statistically significant differences in the mean scores of the authoritative factors related to meeting the DGA for any food category (*p* > 0.05). When examining authoritativeness, significant differences were observed between adolescents who met the DGA criteria and those who did not. Specifically, adolescents who met the DGA had higher scores for authoritarian parenting than those who did not, indicating a positive association between authoritarian parenting and adherence to the DGA. This pattern was consistent across all five food items: fruit (*p* = 0.0013), vegetable (*p* = 0.0101), grain (*p* = 0.0158), dairy (*p* = 0.0016), and protein (*p* = 0.0305). In contrast, no significant differences were found in PSs scores related to setting rules and neglecting, except for dairy consumption, where adolescents who completed the DGA had higher scores for setting rules (*p* = 0.0293) and neglect (*p* = 0.0325) than those who did not.

Adolescents who met the DGA had higher monitoring scores than those who did not, indicating that parental monitoring was associated with better adherence to the guidelines. This trend was also observed for fruit (*p* = 0.0095), vegetable (*p* = 0.0311), grain (*p* = 0.0083), and dairy (*p* = 0.0003). In addition, adolescents who met the DGA criteria had higher scores on reasoning and copying FPPs than those who did not meet the DGA criteria for all MyPlate items, except vegetables (fruit: *p* = 0.0006 and *p* = 0.0247; grain: *p* = 0.0008 and *p* = 0.0059; dairy: *p* = 0.0009 and *p* = 0.0004; protein: *p* = 0.0184 and *p* = 0.0467, respectively for reasoning and copying). Adolescents who met the DGA exhibited higher modeling scores for fruit and dairy consumption than those who did not meet the DGA (fruit: *p* = 0.0222; dairy: *p* = 0.0007).

Next, a stepwise logistic regression was performed for each MyPlate food item to examine the relationship between meeting the DGA-recommended DMA of food item consumption and PSs and FPPs scores, while controlling for confounders. Details of the analyses are presented in Table 5.

**Table 5:** Relationship between meeting the DGA-recommended DMA of the frequency of consumption of MyPlate food items and PSs and FPPs while controlling for cofounders.

	Reference	Estimate (SE)	Odd Ratio (95% Cl)	*p*-value
Fruit				
Authoritative		−1.24 (0.32)	0.29 (0.15–0.54)	0.0001
Authoritarian		0.55 (0.20)	1.73 (1.77–2.54)	0.0054
Monitoring		0.75 (0.29)	2.12 (1.20–3.73)	0.0091
Reasoning		0.77 (0.28)	2.16 (1.20–3.73)	0.0055
Sex	Female^1^	0.51 (0.17)	2.76 (1.41–5.41)	0.0031
Education	Above high school^2^	0.44 (0.18)	2.40 (1.17–4.92)	0.0167
Vegetable				
Authoritarian		0.43 (0.17)	1.54 (1.10–2.15)	0.0117
Setting rules		−0.69 (0.22)	0.50 (0.32–0.78)	0.0019
Monitoring		0.63 (0.21)	1.87 (1.24–2.82)	0.0027
Marital status	Married^3^	0.31 (0.15)	1.87 (1.03–3.38)	0.0394
Grain				
Authoritarian		0.31 (0.17)	1.36 (0.97–1.91)	0.0743
Setting rules		−0.57 (0.23)	0.56 (0.36–0.89)	0.0134
Reasoning		0.75 (0.22)	2.13 (1.37–3.31)	0.0008
Dairy	Reference	Estimate (SE)	Odd Ratio (95% Cl)	*p*-value
Monitoring		0.49 (0.22)	1.63 (1.06–2.50)	0.0193
Copying		0.51 (0.24)	1.66 (1.04–2.65)	0.0260
Sex	Female	−0.38 (0.16)	0.47 (0.25–0.88)	0.0321
Protein	Reference	Estimate (SE)	Odd Ratio (95% Cl)	*p*-value
Authoritarian		0.36 (0.21)	1.43 (0.94–2.17)	0.0912
Setting rules		−0.47 (0.26)	0.62 (0.37–1.04)	0.0706
Reasoning		0.57 (0.25)	1.77 (1.07–2.92)	0.0249
Education	Above high school	0.40 (0.19)	2.24 (1.05–4.75)	0.0363

1. Female vs male; 2. Above high school vs below high school; and 3. Married vs single.CI, confidence interval; DGA, Dietary Guidelines for Americans; DMA, Daily Minimum Amount; FPPs, food-related parenting practices; PS, parenting style.

The results revealed that authoritative (*p* = 0.0001), authoritarian (*p* = 0.0054), monitoring (*p* = 0.0091), reasoning (*p* = 0.0055), adolescent sex (*p* = 0.0031), and parental education (*p* = 0.0167) were significantly correlated with the consumption of the DGA-recommended DMA of fruit among adolescents. For every one score increase in authoritarian PS, monitoring and reasoning FPP, adolescents were approximately 1.7, 2.1, and 2.2 times more likely to consume the DGA-recommended DMA of fruits, respectively. However, for every one-score increase on the authoritative PS, African-American adolescents were 71% less likely to consume the DGA-recommended DMA of fruits. Female adolescents were 2.8 times more likely to consume the DGA-recommended DMA of fruit than male adolescents. Moreover, adolescents with parents with an educational attainment of high school or higher were 2.4 times more likely to consume the DGA-recommended DMA of fruit.

The analysis of vegetable consumption revealed that authoritarian (*p* = 0.0117), setting rules (*p* = 0.0019), monitoring (*p* = 0.0027), and marital status (*p* = 0.0394) were significantly correlated with consuming the DGA-recommended DMA of vegetables among African-American adolescents. African-American adolescents with one higher score in the setting rules style were 50% less likely to consume the DGA-recommended DMA of vegetables. However, for every one-score increase in authoritarian PS and monitoring FPP, adolescents were approximately 1.5 and 1.9 times more likely to consume the DGA-recommended DMA of vegetables. In addition, adolescents living in households with both parents were 1.9 times more likely to consume the DGA-recommended DMA of vegetables.

The results of the regression analysis for grain consumption revealed that the setting rules PS (*p* = 0.0134) and reasoning FPP (*p* = 0.0008) were significantly correlated with the consumption of the DGA-recommended DMA of grains. Based on these results, for every one-score increase in reasoning, adolescents were approximately 2.1 times more likely to consume the DGA-recommended DMA of grains, whereas adolescents with one score higher in setting rules were approximately 44% less likely to consume the recommended amount.

The results of the regression analysis for dairy consumption showed that monitoring (*p* = 0.0193), copying FPP (*p* = 0.0260), and sex (*p* = 0.321) were significantly correlated with the consumption of the DGA-recommended DMA of dairy among adolescents. Based on these results, for every one-score increase in monitoring and copying FPPs, adolescents were approximately 1.6 and 1.7 times more likely to consume the DGA-recommended DMA of dairy, respectively. Furthermore, females were 53% less likely than males to meet the DGA for this food group.

The results of the regression analysis for protein consumption showed that reasoning (*p* = 0.0249) and parental education (*p* = 0.0363) were significantly correlated with consumption of the DGA-recommended DMA of protein among adolescents. For every one-score increase in reasoning and having parents with educational attainment of high school or higher, adolescents were 1.8 and 2.4 times more likely to consume the DGA-recommended DMA of protein, respectively.

## 4 Discussion

The aim of this study was to assess the impact of PSs and FPPs on the adherence of African-American adolescents to the DGA. Specifically, we focused on determining which PSs and FPPs impact the consumption of recommended DMA across various MyPlate food categories. In relation to different PSs, an authoritative PS has been reported as an effective strategy to raise children with healthy eating habits and normal weight status (17, 29, 32). However, we found that adolescents with an authoritative PS did not consume the DGA-recommended DMA of MyPlate food items. This discrepancy can be attributed to the differences in the race/ethnicity of the samples in the studies, underscoring the necessity for culturally tailored parenting strategies. According to our study, African-American adolescents with an authoritative PS were less likely to consume the DGA-recommended DMA of fruit. This indicates that a higher score on authoritative PS is not a suitable tactic for increasing fruit consumption to the DMA among African-American adolescents. Setting rules plays a role similar to that of the authoritative style in ensuring the consumption of DGA-recommended amounts of vegetables and grains. This is consistent with the results of a previous study revealing that rules can have a negative impact on children’s inability to regulate their food intake (33). However, setting at least one health-oriented food rule at home is directly associated with healthy, independent eating choices among early adolescents (30). Setting rules may be a good strategy for younger adolescents, but plays a negative role in threatening the autonomy of older adolescents. However, although the authoritarian style is characterized by extreme control and obedience, it plays a positive role in ensuring the consumption of DGA-recommended DMA in fruits and dairy. The findings of our study indicate that, among African-American adolescents, those who experienced a higher degree of authoritarian parenting were more likely to meet the DGA recommendations for fruit and vegetable consumption. This suggests that strict rules and discipline associated with authoritarian parenting may play a positive role in promoting healthier eating habits in this population. It is worth noting that previous studies examining parenting styles and dietary behaviors have mostly been conducted in the general population, which may explain the inconsistencies between our findings and those of previous research. By focusing specifically on African-American adolescents, our study provides valuable insights into the unique influences on dietary behaviors within this demographic group.

Among the four FPPs, monitoring and reasoning emerged as strategies that parents could adopt to ensure their adolescents consume the DGA-recommended DMA for at least three MyPlate food items. Adolescents who experienced monitoring FPP met the DGA for fruit, vegetables, and dairy, while adolescents with higher reasoning FPP met the DGA for fruit, grain, and protein consumption. Monitoring FPP had the largest positive impact on fruit consumption and the smallest on dairy, according to the DGA. Our previous study, which involved a smaller sample size, examined the same PSs and FPPs also confirmed a positive correlation between monitoring FPP and an increase in fruit and vegetable consumption and a decrease in unhealthy snack consumption among African-American adolescents (13, 18). Beckers et al. reported a curvilinear relationship between monitoring and adolescent eating behavior, indicating that monitoring can promote healthy eating to a certain extent; however, extreme monitoring has a negative role in the evolution of an unhealthy diet among adolescents (34). Thus, considering the level of age-suitable independence is important to adjust the optimal level of FPP monitoring. Furthermore, reasoning FPP had the largest positive impact on meeting the DGA for fruit consumption and the smallest positive impact on meeting the DGA for protein consumption. Reasoning practice is defined as a tool used to convince adolescents to adopt better eating behaviors by transmitting nutritional knowledge (35). Enhancing this kind of knowledge promotes autonomy among adolescents, which can impact their self-efficacy regarding the foods they consume. Additionally, copying emerged as another FPP in which parents encouraged their children to imitate their own eating behaviors, intentionally or unintentionally (30). The findings of this study suggest that copying behavior also influences whether adolescents meet the recommended guidelines for dairy consumption. Recognizing the role of copying can help develop strategies to promote positive parental behaviors and foster healthier dairy consumption habits among adolescents.

Furthermore, along with PSs and FPPs, specific demographic factors influenced the attainment of the DGA-recommended DMA for MyPlate food items. Sex plays a significant role in meeting the DGA for fruit consumption, with females having a higher likelihood of meeting the DGA-recommended amounts than males. However, females were less likely to meet the DGA for dairy consumption than males. This can be attributed to various factors, including differences in taste preferences, dietary choices, and cultural norms. Moreover, residing in educated African-American households was associated with an increased probability of consuming DGA-recommended amounts of fruit and protein. Higher levels of education among parents or caregivers may lead to greater awareness and understanding of nutrition, thereby promoting healthier food choices for adolescents. Education serves as a pathway for acquiring knowledge about the impact of nutrition on health, empowering individuals to make informed decisions about their diet. Moreover, our study revealed that adolescents living in married households were more likely to meet the DGA-recommended vegetable consumption. This finding suggests that a stable marital environment creates a supportive atmosphere that encourages healthy dietary habits among adolescents. Shared mealtime practices and a family environment that values and promotes vegetable consumption may contribute to an increased likelihood of meeting the DGA guidelines.

One strength of this study was the use of all five MyPlate food groups, whereas previous studies considered only one or two food groups to examine the impact of PSs/FPPs. By including all five MyPlate food items, our study offers a comprehensive evaluation of dietary patterns, providing a holistic view of the impact of studied parenting on dietary adherence among African-American family. In this study, PSs and FPPs were considered together to determine which among them plays an important role in the diet of African-American adolescents. To our knowledge, this is the first study to use information on the consumption of the DGA-recommended DMA to determine the effectiveness of PSs and FPPs in eliciting behaviors toward the consumption of different food items. The participation of the parent–adolescent dyad is another strength of this study, which reduced the bias from collecting data from just one participant. Additionally, we focused on a minority group, African-Americans, who have been less studied.

However, this study had certain limitations. The data collection to assess dietary habits was based on the FFQ, which, while practical and widely used, is subject to recall bias, as it relies on the participant’s memory of their dietary intake. Additionally, the fixed list of food items may not capture the full diversity of the adolescents’ diet, especially for those with unique eating habits. This can lead also to under- or over-reporting of data, which can directly impact the results. Future studies should use 24 h recalls combined with an FFQ survey to increase the accuracy of dietary intake data collection. Moreover, additional FPPs, such as the availability and accessibility of food, rules, and expectations, as well as pressure-to-eat or the effect of parenting styles, including permissive and neglectful styles, can be included in future studies to comprehensively analyze parenting practices. Finally, studies on macro- and micronutrient components can be included in the food consumption analysis. In this study, we considered the total grain (refined and whole grains) consumption. Therefore, these results can be attributed to a higher proportion of refined grains than whole grains. Most Americans consume adequate amounts of total grain food, whereas few consume adequate amounts of whole grains (36). Future studies should consider these two types of grains separately to obtain more detailed results.

Finally, this study highlights the significant role of PSs and FPPs in shaping the dietary behaviors of African-American adolescents. This underscores the importance of PSs and FPPs in meeting the DGA recommendations, particularly for different food categories. These findings suggest that specific PSs, FPPs, and certain demographic factors are associated with increased or decreased odds of meeting DGA criteria for various food items. These insights contribute to a better understanding of suitable parenting approaches within a cultural context and can positively influence the eating behaviors of adolescents, ultimately improving the health of families and communities.

## Data availability statement

The raw data supporting the conclusions of this article will be made available by the authors, without undue reservation.

## Ethics statement

This research study was approved by the Institutional Review Board at the University of the District of Columbia (IRB#878591-2) on 16 February 2021. The studies were conducted in accordance with the local legislation and institutional requirements. Written informed consent for participation in this study was provided by the participants’ legal guardians/next of kin.

## Author contributions

AA: Conceptualization, Investigation, Methodology, Visualization, Writing – original draft, Writing – review & editing. LM-L: Conceptualization, Funding acquisition, Writing – original draft, Writing – review & editing. DW: Formal analysis, Methodology, Validation, Writing – review & editing. CC: Conceptualization, Methodology, Supervision, Validation, Writing – review & editing.
